# Light Emission from the Fe^2+^-EGTA-H_2_O_2_ System: Possible Application for the Determination of Antioxidant Activity of Plant Phenolics

**DOI:** 10.3390/molecules23040866

**Published:** 2018-04-10

**Authors:** Michal Nowak, Wieslaw Tryniszewski, Agata Sarniak, Anna Wlodarczyk, Piotr J. Nowak, Dariusz Nowak

**Affiliations:** 1 Radiation Protection, University Hospital No 2, Medical University of Lodz, Zeromskiego 113, 90-549 Lodz, Poland; m.nowak@skwam.lodz.pl; 2 Department of Radiological and Isotopic Diagnostics and Therapy, Medical University of Lodz, Zeromskiego 113, 90-549 Lodz, Poland; wieslaw.tryniszewski@umed.lodz.pl; 3 Department of General Physiology, Medical University of Lodz, Mazowiecka 6/8, 92-215 Lodz, Poland; agata.sarniak@umed.lodz.pl; 4 Department of Sleep Medicine and Metabolic Disorders, Medical University of Lodz, Mazowiecka 6/8, 92-215 Lodz, Poland; anna.wlodarczyk@umed.lodz.pl; 5 Department of Nephrology, Hypertension, and Kidney Transplantation, Medical University of Lodz, Pomorska 251, 92-213 Lodz, Poland; piotr.nowak@umed.lodz.pl; 6 Department of Clinical Physiology, Medical University of Lodz, Mazowiecka 6/8, 92-215 Lodz, Poland

**Keywords:** chemiluminescence, Fenton system, plant phenolic acids, antioxidant activity

## Abstract

Oxidative reactions can result in the formation of electronically excited species that undergo radiative decay depending on electronic transition from the excited state to the ground state with subsequent ultra-weak photon emission (UPE). We investigated the UPE from the Fe^2+^-EGTA (ethylene glycol-bis(β-aminoethyl ether)-*N*,*N*,*N*′,*N*′-tetraacetic acid)–H_2_O_2_ system with a multitube luminometer (Peltier-cooled photon counter, spectral range 380 to 630 nm). The UPE of 92.6 µmol/L Fe^2+^—185.2 µmol/L EGTA—2.6 mmol/L H_2_O_2_ reached 4319 ± 755 relative light units during 2 min measurement and was about seven times higher (*p* < 0.001) than the UPE of incomplete systems (Fe^2+^-H_2_O_2_, EGTA-H_2_O_2_) and medium alone. Substitution of Fe^2+^ with Cr^2+^, Co^2+^, Mn^2+^ or Cu^2+^ as well as of EGTA with EDTA (ethylenediaminetetraacetic acid) or citrate completely abolished UPE. Experiments with ROS scavengers revealed the dependence of UPE on hydroxyl radicals suggesting occurrence of oxidative attack and cleavage of the ether bond in EGTA backbone structure and formation of triplet excited carbonyl groups with subsequent light emission. Plant phenolics (ferulic, chlorogenic and caffec acids) at concentration 87 µmol/L and ascorbate at 0.46 mmol/L inhibited UPE by 90 ± 4%, 90 ± 5%, 97 ± 2% and 92 ± 1%, respectively. Quenching of UPE from Fe^2+^-EGTA-H_2_O_2_ system can be used for evaluation of antioxidant activity of phytochemicals.

## 1. Introduction

Oxidative metabolic reactions in living cells can result in the formation of electronically excited species. They undergo radiative decay depending on electronic transition from the singlet or triplet excited state to the singlet ground state what is accompanied by ultra-weak photon emission (UPE) [1]. In vitro oxidation of pure lipids or cell cultures and organ homogenates containing lipids (especially brain homogenates) resulted in UPE along with accumulation of various products of lipid peroxidation [2,3,4,5,6]. Free radical scavengers (mannitol, butylated hydroxytoluene, D-α-tocopherol) quenched UPE related to lipid peroxidation in cell culture and organ homogenates [3,4]. Moreover, rats fed a tocopherol-free diet for seven months revealed higher ex vivo UPE from brain, liver and heart homogenates than animals on normal feed [4]. Fenton’s reagent (solution of H_2_O_2_ with Fe^2+^) and chelate-modified Fenton’s reagent are used to study the hydroxyl radical (^•^OH)-induced peroxidative damage to various organic compounds and biomolecules and in some cases this can be accompanied by light emission including UPE [7,8,9]. Because, the Fenton reaction involves the creation of reactive oxygen species (ROS) by chemicals that are present in vivo these experimental models have importance in studies on free radicals related pathology in humans [10,11]. Moreover, measurement of UPE as well as other forms of chemiluminescence related to peroxidative damage to biomolecules can be used for monitoring the effectiveness of various compounds as potential ROS scavengers and pharmacological interventions leading to suppression of oxidative stress in vivo [12,13]. In addition, chemiluminescence can reflect the intensity of oxidative processes and current balance between generated ROS and antioxidant capacity in various cells and tissues under normal conditions [1,14].

A recent study showed that chelating agents frequently used to modify Fenton’s reagent such as EDTA and EGTA can react with various oxidants including hypochlorite, peroxyl radicals and peroxynitrite [15]. It cannot be excluded that these reactions lead to generation of electronically excited chemical groups in chelating compounds with subsequent light emission. Although, Fenton’s reagent alone was reported to generate UPE [7,8] no data exist (to the best of our knowledge) on photon emission from Fe^2+^-EGTA-H_2_O_2_ system. Therefore, in this study we investigated the chemilumiescence of Fe^2+^-EGTA-H_2_O_2_ system with special attention to elucidate what ROS are involved in this phenomenon and its possible application as a tool for evaluation of antioxidant activity of selected phenolic acids.

## 2. Results 

### 2.1. Ultra Weak Photon Emission from Fe^2+^-EGTA-H_2_O_2_ System 

The complete system 9.3 µmol/L Fe^2+^—18.5 µmol/L EGTA—0.26 mmol/L H_2_O_2_ emitted 1351 ± 178 relative light units (RLU) during 2 min of measurement. This was about 2.2-times higher (*p* < 0.001) than UPE from incomplete systems (Fe^2+^-H_2_O_2_ or EGTA-H_2_O_2_) and 2.5-times higher (*p* < 0.001) than that of medium alone (Table 1). 

Analysis of 5-, 10- and 20-times higher concentrations of Fe^2+^-EGTA-H_2_O_2_ system (under conditions of the same ratio of molar concentrations of constituents) revealed gradual increase in UPE from 1533 ± 76 RLU up to 6278 ± 502 RLU while light emission from control systems (incomplete systems, H_2_O_2_ alone, Fe^2+^-EGTA-H_2_O) did not change significantly and ranged from 489 ± 9 RLU to 645 ± 100 RLU (Table 1). This increase was not linear and the difference between mean UPE of Fe^2+^-EGTA-H_2_O_2_ and mean background photon emission of medium alone (H_2_O injected to PBS) was 821 RLU, 1061 RLU, 3718 RLU and 5781 RLU for the baseline and 5-, 10- and 20-times higher concentrations of the modified Fenton system, respectively. It should be pointed out that light emission from Fe^2+^-H_2_O_2_ (incomplete system I, Fenton reagent) in the case of two highest concentrations (experiment C and D, Table 1) was higher (p < 0.05) than those from corresponding control systems. The UPE of 92.6 µmol/L Fe^2+^-185.2 µmol/L EGTA-2.6 mmol/L H_2_O_2_ reached 4319 ± 755 RLU and was about seven times higher than UPE of incomplete systems and medium alone (Table 1). This concentration of Fenton system was used for further experiments.

### 2.2. Effect of Iron and EGTA Replacement by other Divalent Cations and Metal Chelators on Light Emission from Fe^2+^-EGTA-H_2_O_2_ System

**Figure 1:**
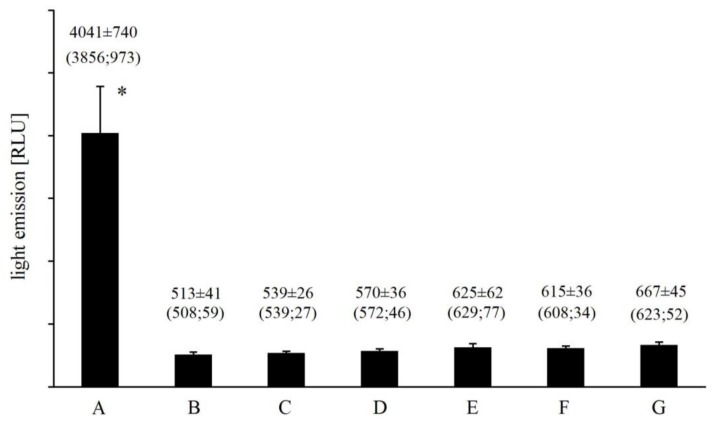
Effect of iron and EGTA replacement with other divalent cations (Cu^2+^, Mn^2+^, Co^2+^, Cr^2+^) and metal chelators (EDTA, citric acid) on the light emission from Fe^2+^-EGTA-H_2_O_2_ system. Results obtained from four series of experiments expressed as mean and standard deviation and (median; interquartile range). (A) Fe^2+^-EGTA-H_2_O_2_; (B) Cu^2+^-EGTA-H_2_O_2_; (C) Mn^2+^-EGTA-H_2_O_2_; (D) Co^2+^-EGTA-H_2_O_2_; (E) Cr^2+^-EGTA-H_2_O_2_; (F) Fe^2+^-EDTA-H_2_O_2_; (G) Fe^2+^-citric acid-H_2_O_2_. * vs. value of B, C, D, E, F and G, *p* < 0.05.

Replacement of Fe^2+^ with other divalent cations (Cr^2+^, Co^2+^, Mn^2+^, Cu^2+^) almost completely abolished any light emission from the Fenton system (Figure 1). Mean UPE ranged from 513 ± 41 RLU for Cu^2+^-EGTA-H_2_O_2_ to 625 ± 62 RLU for Cr^2+^-EGTA-H_2_O_2_ and was comparable to that observed for medium alone (508 ± 28 RLU). Similar results were observed for Fe^2+^-EDTA-H_2_O_2_ and Fe^2+^-citric acid-H_2_O_2_, both systems emitted 6.5- and 6-times less photons (Figure 1) than Fe^2+^-EGTA-H_2_O_2_ over 2 min of counting, respectively. Mean photon emission from all corresponding control systems did not exceed 690 RLU (data not shown). 

### 2.3. Effect of Reactive Oxygen Species Scavengers on Light Emission from Fe^2+^-EGTA-H_2_O_2_ System

**Figure 2:**
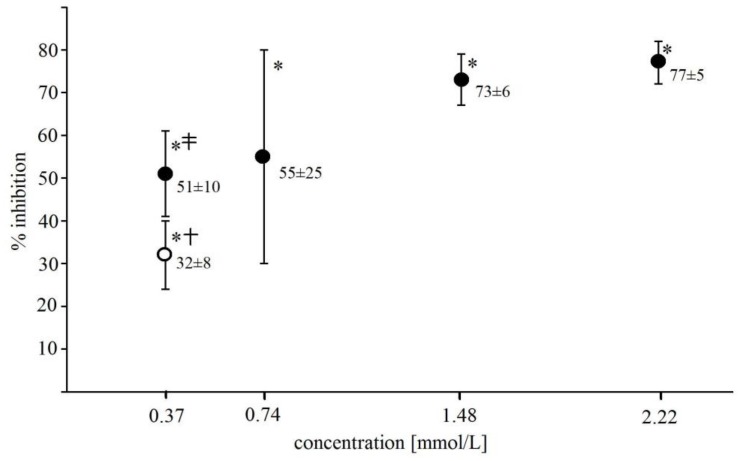
Inhibitory effect of DMSO (closed circles) and mannitol (open circle) on light emission from 92.6 µmol/L Fe^2+^—185.2 µmol/L EGTA—2.6 mmol/L H_2_O_2_ system. DMSO and mannitol were added to PBS containing Fe^2+^ and EGTA before automatic H_2_O_2_ injection. Results expressed as mean and standard deviation of % inhibition were obtained from 6 separate experiments. * significant inhibition, *p* < 0.001; † vs. corresponding concentration of DMSO, *p* < 0.05; ‡ vs. DMSO concentrations of 1.48 mmol/L and 2.22 mmol/L, *p* < 0.01.

Catalase and superoxide dismutase (SOD) added to Fe^2+^-EGTA (final activity of 0.185 U/µL) prior to a H_2_O_2_ injection inhibited UPE by 85 ± 4% and 65 ± 14%, respectively (*n* = 8, *p* < 0.001). NaN_3_ (an singlet oxygen scavenger) at the concentration of 0.37 mmol/L had no significant effect on UPE of Fe^2+^-EGTA-H_2_O_2_ (4521 ± 441 RLU for Fenton system without NaN_3_ vs. 3784 ± 1027 RLU for system with NaN_3_, *p* > 0.05, *n* = 6) while hydroxyl radical (^•^OH) scavengers, mannitol and dimethyl sulfoxide (DMSO) at the same concentration decreased UPE by 32 ± 8% and 51 ± 10% (*p* < 0.01), respectively, with the stronger effect of the latter one (*p* < 0.05) (Figure 2). 

### 2.4. Effect of Selected Phenolics and Ascorbic Acid on Light Emission from Fe^2+^-EGTA-H_2_O_2_ System

All tested phenolic acids (ferulic, chlorogenic and caffec acids) strongly suppressed the light emission from Fe^2+^-EGTA-H_2_O_2_ system. Addition of ferulic acid, chlorogenic acid or caffeic acid to the final concentration of 87 µmol/L decreased the mean UPE from Fe^2+^-EGTA-H_2_O_2_ by 4.7-, 4.5- and 7.7-times (*p* < 0.01), respectively. The mean % inhibition of light emission ranged between 90 ± 3% and 98 ± 1% for these compounds at the concentration range of 87 µmol/L to 870 µmol/L and the strongest inhibition was observed in the case of caffeic acid (Table 2). Ascorbic acid at the concentration of 0.46 mmol/L almost completely quenched UPE from Fe^2+^-EGTA-H_2_O_2_ (92 ± 1% inhibition, *n* = 4, *p* < 0.001). However, the light emission from Fe^2+^-EGTA-ascorbic acid-H_2_O_2_ was still higher than that of medium alone (963 ± 44 RLU vs. 585 ± 8 RLU, *p* < 0.05). 

## 3. Discussion

### 3.1. Light Emission from Fe^2+^-EGTA-H_2_O_2_ System

We found that Fe^2+^-EGTA-H_2_O_2_ system emitted light in a concentration dependent (but not linear) manner under conditions of a stable ratio of molar concentrations of its constituents. Injection of H_2_O_2_ to Fe^2+^ or EGTA alone did not result in the significant increase in UPE. In these cases the light emission was similar or slightly higher than that observed for medium alone. Substitution of Fe^2+^ with other divalent cations (Cu^2+^, Mn^2+^, Co^2+^ and Cr^2+^) almost completely abolished UPE. The same effect was observed for substitution of EGTA with EDTA or citric acid. These indicate that UPE is specific for Fe^2+^-EGTA-H_2_O_2_ system and could not be obtained from the combination of other divalent cations and chelating agents with H_2_O_2_.

### 3.2. Plausible Mechanism of Light Generation from Fe^2+^-EGTA-H_2_O_2_ System

Light generation from Fe^2+^-EGTA-H_2_O_2_ system was inhibited by ^•^OH scavengers (mannitol and DMSO), SOD an effective scavenger of superoxide radical (O_2_^−^) and catalase an enzyme decomposing H_2_O_2_. Inhibitory effect of catalase on UPE is obvious and clearly indicates the necessity of H_2_O_2_ for light emission and is in line with the lack of UPE from incomplete system Fe^2+^-EGTA-H_2_O. Numerous reactions can simultaneously take place in our modified Fenton system. Some of them leading to generation of ^•^OH, O_2_^−^ and singlet oxygen (O_2_(^1^Δ_g_)) [7,8,16] are shown below:

Fe^2+^-EGTA + H_2_O_2_ → Fe^3+^-EGTA + OH^−^ + ^•^OH (hydroxyl radicals generation) (1)

Fe^3+^-EGTA + H_2_O_2_ → Fe^3+^OOH^−^-EGTA + H^+^ (2)

Fe^3+^OOH^−^-EGTA + H_2_O_2_ → FeO^2+^-EGTA + HO^•^_2_ + H_2_O (3)

FeO^2+^-EGTA + H_2_O_2_ → Fe^3+^-EGTA + HO^•^_2_ + OH^−^ (4)

HO^•^_2_ → H^+^ + O_2_^−^ (formation of superoxide radicals) (5)

O_2_^−^ + Fe^3+^-EGTA → Fe^2+^-EGTA + O_2_ (reduced iron can enter reaction 1 to yield ^•^OH) (6)

O_2_^−^ + ^•^OH + H^+^ → H_2_O_2_ + O_2_(^1^Δ_g_) (formation of singlet oxygen) (7)

2 O_2_^−^ + 2 H+ → H_2_O_2_ + O_2_(^1^Δ_g_) (formation of singlet oxygen) (8)

It is well known that Fenton reagent (Fe^2+^-H_2_O_2_) generates UPE via O_2_(^1^Δ_g_) formation and its decay [7,8] with a three characteristic bands emission at 1270 nm (monomolecular decay from its first excited state to ground state), and at 634 nm and 703 nm (bimolecular transition) [17]. Two of these bands (1270 nm and 703 nm) were far away and one (634 nm) was at the border of the spectral range (from 380 nm to 630 nm) of detection of our luminometer. The width of the 634 nm band is about 35 nm, therefore this may be at least in part responsible for UPE of Fe^2+^-EGTA-H_2_O_2_ under conditions of our experiments. However, NaN_3_ a scavenger of O_2_(^1^Δ_g_) did not significantly decrease UPE which suggests that O_2_(^1^Δ_g_) was not involved in UPE of Fe^2+^-EGTA-H_2_O_2_ system. There are two possible explanations of this observation: firstly, the intensity of photons emission related to bimolecular decay of O_2_(^1^Δ_g_) was too low to significantly contribute to UPE of Fe^2+^-EGTA-H_2_O_2_ system and was not detected; secondly, almost all O_2_·^−^ (generated in reaction 5) was consumed for reduction of Fe^3+^-EGTA complex (reaction 6) and thus formation of O_2_(^1^Δ_g_) and subsequent photons emission was inhibited. Another source of UPE are triplet excited carbonyl groups (^3^(R=C)*) [2,18,19] emitting photons with spectral range of 350 nm to 550 nm [12]. 

**Figure 3:**
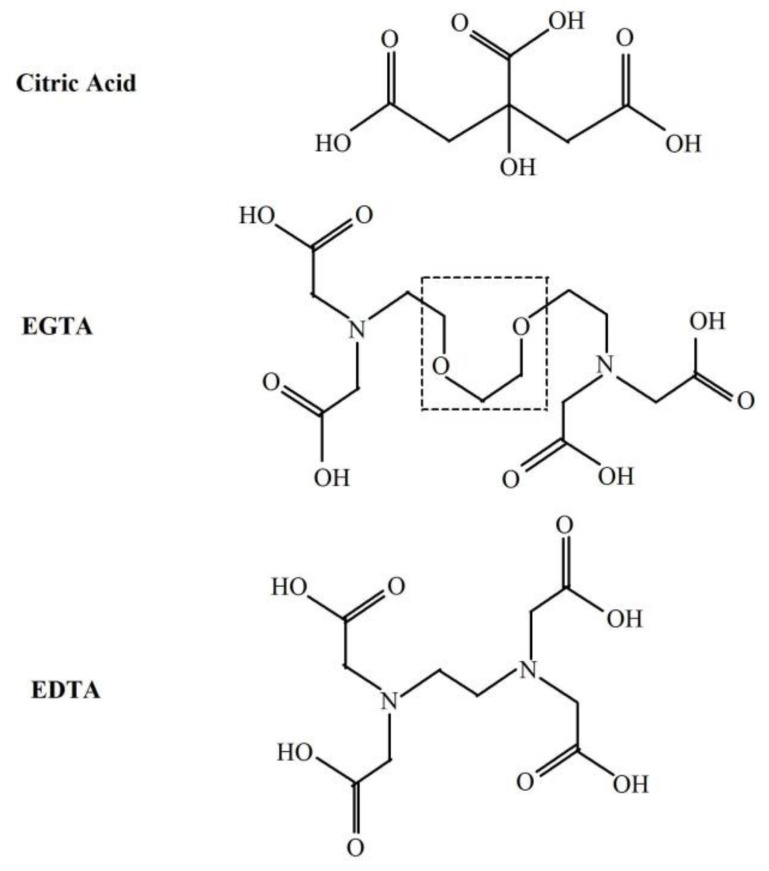
Chemical structures of citric acid, EGTA and EDTA. The dashed line frame shows the two ether bonds of EGTA most probably involved in the light emission from the Fe^2+^-EGTA-H_2_O_2_ system.

**Figure 4:**
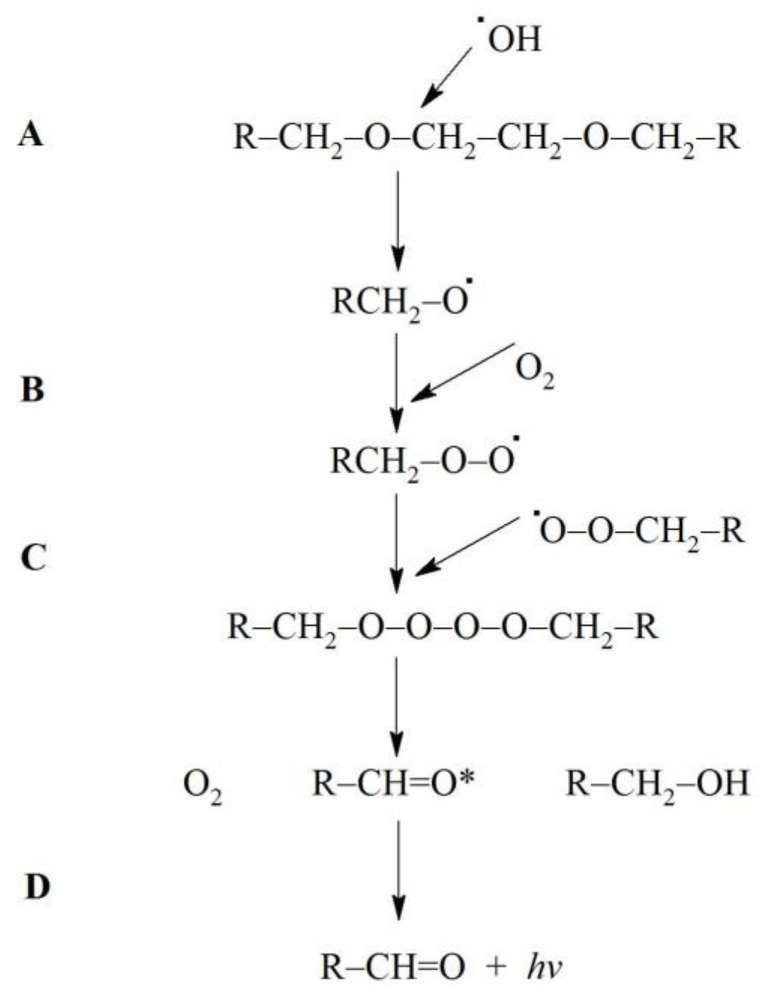
The proposed mechanism for formation of triplet excited carbonyl groups (^3^(R=C)*) and subsequent light emission from Fe^2+^-EGTA-H_2_O_2_ system. (A) Hydroxyl radicals (^•^OH) generated in the Fenton reaction attack one of ether bond in the backbone structure of EGTA (R-CH_2_-O-CH_2_-CH_2_-O-CH_2_-R) leading to its cleavage and radicals formation (R-CH_2_-O^•^). (B) This radical react with molecular oxygen (O_2_) dissolved in reaction environment to generate peroxyl radical(R-CH_2_-O-O^•^). (C) Two peroxyl radicals react with each other (Russel-type mechanism) with subsequent formation of O_2_, and two products one with hydroxyl group (R-CH_2_-OH) and the second one with triplet excited carbonyl group (R-CH=O*). (D) Electronic transitions from the triplet excited state to the ground state is accompanied by the photon emission (λν).

^3^(R=C)* can be formed by ^•^OH—induced oxidation of various low molecular weight compounds (e.g., uric acid, vitamin B_12_, tryptophan) as well as lipids and DNA [12]. ^•^OH can attack the ether group in the backbone chain of various molecules resulting in the cleavage of the ether bond [20,21,22] with its further degradation and formation of another radical and carbonyl group [20,23]. EGTA contains two ether bonds in the middle of the backbone chain (Figure 3). It is possible that ^•^OH can react with these bonds and form ^3^(R=C)* with subsequent photons emission. Figure 4 shows the proposed mechanism of these reactions. This suggested elucidation of light emission from Fe^2+^-EGTA-H_2_O_2_ system is supported by three observations: (A)—substitution of EGTA with EDTA which has no ether bonds in the backbone structure (Figure 3) resulted in the elimination of UPE; (B)—^•^OH scavengers inhibited UPE probably by protection of EGTA ether bonds from ^•^OH attack and consequent formation of ^3^(R=C)*; (C)—SOD decreased UPE by scavenging O_2_^−^ and suppression of ^•^OH formation via inhibition of reaction (6). This reaction in the presence of SOD is as follows:

O_2_^−^ + 2H^+^ + Fe^3+^-EGTA → Fe^3+^-EGTA + H_2_O_2_ (no reduction of Fe^3+^ and subsequent ^•^OH formation) (9)

Moreover, substitution of EGTA with citric acid (other chelating agent without ether bond) also abolished UPE.

### 3.3. Inhibitory Effect of Phenolic Acids on Light Emission from Fe^2+^-EGTA-H_2_O_2_ System

According to the proposed mechanism of UPE from Fe^2+^-EGTA-H_2_O_2_ any given compound would inhibit light emission if: (A) it effectively scavenges at least one of the following ROS: H_2_O_2_, ^•^OH and O_2_^−^; (B) is a stronger Fe^2+^ chelating agent than EGTA. The first action would result in the decreased activity of ^•^OH and protection of EGTA ether bonds from oxidative attack. The second one would involve abstraction of Fe^2+^ ions from Fe^2+^-EGTA complex and formation of another complex to be less effective in reaction with H_2_O_2_ leading to ^•^OH formation. Moreover, part of ^•^OH radicals formed in this complex can oxidize other molecules (e.g., added compound with chelating properties or bicarbonate ions derived from dissolved in water atmospheric CO_2_ [24]) before their reaction with ether bonds of EGTA and subsequent light emission.

All three tested phenolic acids inhibited UPE from the Fe^2+^-EGTA-H_2_O_2_ system. They have hydroxyl substituents in the backbone aromatic ring: ferulic acid one, caffeic and chlorogenic acids have two. It is possible that ^•^OH can grab a hydrogen atom from one of the hydroxyl groups at the phenolic ring to form water and a less reactive and more stable radical. Thus less ^•^OH was available for photon emitting reactions with ether bonds of EGTA. The observation that ferulic acid was a weaker inhibitor of UPE than caffeic acid is in line with this explanation. Moreover, this plausible mechanism of UPE inhibition from Fe^2+^-EGTA-H_2_O_2_ system by phenolic acids is in agreement with previous reports demonstrating an intensification of the ^•^OH scavenging activity of flavonoids with an increased number of –OH substituents in an aromatic ring [25]. Furthermore, hydroxyl groups and catechol group, at position 3, 5, 7 and 40 are critical for the effective scavenging of peroxynitrite by flavonoids [26] as well as the inhibition of total ROS generation in kidney homogenates by flavonoids intensifies as the number of total –OH groups in their structure increases [27]. Moreover, the protective effect of polyphenols against ^•^OH—induced degradation of deoxyribose correlated with the number of –OH substitutions in the backbone structure [28]. Our results correspond well with a studies showing distinct ^•^OH and O_2_·^−^ scavenging activity of chlorogenic and caffeic acids in vitro [29] and the inhibitory effect of ferulic acid on ^•^OH—induced damage to synaptosomes and neuronal cells [30,31]. In another study ferulic acid scavenged O_2_·^−^ as proved by using electron spin resonance spectroscopy [32]. Therefore, decomposition of O_2_·^−^ apart from direct scavenging of ^•^OH may additionally be responsible for inhibitory effect of studied phenolic acids on UPE from Fe^2+^-EGTA-H_2_O_2_ system. These phenolic acids can also from complexes with divalent cations including Fe^2+^ [33,34,35] which can decrease Fe^2+^ reactivity with H_2_O_2_ [33,34]. However, two-fold molar excess of EGTA compared to Fe^2+^ ions in the reaction mixture seems to prevent formation of Fe^2+^-phenolic acid complexes. It is in line with previous studies showing negligible binding of Fe^2+^ and Fe^3+^ to polyphenols in the presence of excess of EDTA another strong chelating agent [34,36,37]. Therefore, formation of phenolic acid–iron complexes had insignificant contribution to phenolic acid-induced suppression of UPE from Fe^2+^-EGTA-H_2_O_2_. Caffeic, chlorogenic and ferulic acids were able to reduce Fe^3+^ to Fe^2+^ [38]. Thus they can replace O_2_^−^ as an Fe^3+^ reducing agent (reaction 6) and enhance ^•^OH generation in the Fe^2+^-EGTA-H_2_O_2_ system. Therefore although all phenolics revealed about 90% inhibition of UPE at concentration of 87 µmol/L we tested 2- and 10-times higher concentrations to exclude any possible pro-oxidant action of these compounds. Ascorbic acid is also a powerful Fe^3+^ reducing agent [38] and is frequently used as a component of modified Fenton systems (e.g., Fe^3+^-H_2_O_2_-ascorbate and Fe^3+^-EDTA-H_2_O_2_-ascorbate) to enhance ^•^OH generation in in vitro studies on antioxidant properties of various phytochemicals [28]. However, ascorbic acid itself can scavenge various ROS including ^•^OH [39,40] and is recognized as an efficient antioxidant vitamin in vivo [40,41]. Ascorbic acid almost entirely inhibited UPE from Fe^2+^-EGTA-H_2_O_2_ system which shows that scavenging of ^•^OH by this vitamin definitely prevailed over potential pro-oxidant action under conditions of our experiments. 

### 3.4. Strengths and Weaknesses of the Study 

The photon counter of luminometer used for measurement of light emission had narrow spectral range from 380 nm to 630 nm and was cooled with Peltier module only to 8 °C. This precluded the measurement of photons derived from decay of O_2_(^1^Δ_g_) formed in Fenton system (reactions 7 and 8). Application of photomultiplier device with much wider spectral range sensitivity and lower working temperature of photomultiplier (e.g., 300 nm to 900 nm and −40 °C, 160 nm to 710 nm and −30 °C) provides the opportunity to measure signals from all possible photon emitters (^3^(R=C)* and O_2_(^1^Δ_g_)) and ensures low background and high signal-to-noise ratio [2,3]. Therefore, it cannot be excluded that the real UPE from Fe^2+^-EGTA-H_2_O_2_ system is higher than we observed under conditions of our experiments. On the other hand, the AutoLumat Plus is a commercially available instrument for chemiluminescent determination of various compounds and enzymes in one batch in a quasi-parallel mode and our experiments could be easily repeated and extended to various Fenton systems by other researches. Moreover, we proposed the quenching of UPE from Fe^2+^-EGTA-H_2_O_2_ system as a simple tool for evaluation of antioxidant activity of various phytochemicals. Because the system simplicity and short time of UPE recording the assay is inexpensive, suitable for automation and the obtained results could be easy for interpretation. Therefore, a proven possibility to execute such tests with AutoLumat Plus seems to be the advantage of our study. We did not determine spectrum of light emitted from Fe^2+^-EGTA-H_2_O_2_ system and it could also be recognized as the second weakness of our study. However, with the use of various ROS scavengers and by substitution of EGTA with EDTA we were able to conclude that the photons emitters are ^3^(R=C)*. Moreover, by substitution of Fe^2+^ with other divalent cations we proved that UPE emission within the range from 380 nm to 630 nm is specific for Fe^2+^-EGTA-H_2_O_2_ combination. The ratio of molar concentrations of FeSO_4_ to EGTA to H_2_O_2_ in Fenton reaction system was 1:2:28.1. In our previous studies this reaction mixture generated large amounts of ^•^OH as reflected by damage to deoxyribose [28,42] and the cytotoxicity against cell suspensions in vitro [43]. Therefore, we analyzed the relationship between UPE and increasing concentrations of Fenton system under conditions of stable ratio of molar concentrations of its components. The maximal photon emission from Fenton system occurred during the first several dozen seconds after addition of H_2_O_2_ to Fe^2+^ solution and then was terminated [8]. Therefore, we measured UPE from Fe^2+^-EGTA-H_2_O_2_ system for 120 s and did not analyze the kinetics of this phenomenon. 

## 4. Materials and Methods

### 4.1. Reagents

All chemicals were of analytical grade. DMSO, d-mannitol, sodium azide (NaN_3_), iron (II) sulfate heptahydrate (FeSO_4_·7H_2_O), cupric sulfate pentahydrate (CuSO_4_·5H_2_O), cobalt (II) sulfate hydrate (CoSO_4_·H_2_O), manganese (II) sulfate monohydrate (MnSO_4_·H_2_O), chromium (II) chloride (CrCl_2_), EDTA, EGTA, sodium citrate, ferulic, chlorogenic and caffeic acids (see Appendix A), sodium L-ascorbate, catalase from bovine liver (2440 units/mg solid), SOD from bovine liver (1500 units/mg protein) were purchased from Sigma-Aldrich Chemical (St. Louis, MO, USA). H_2_O_2_ 30% solution (*w*/*w*) was from Chempur (Piekary Slaskie, Poland). Sterile phosphate buffered saline (PBS, pH 7.4, without Ca^2+^ and Mg^2+^) was obtained from Biomed (Lublin, Poland). Sterile deionized pyrogen-free water (freshly prepared, resistance > 18 MW/cm, HPLC H_2_O Purification System, USF Elga, Buckinghamshire, UK) was used throughout the study. Working aqueous solutions of FeSO_4_ (concentrations of 0.5, 2.5, 5, and 10 mmol/L) and 5 mmol/L solutions of CuSO_4_, MnSO_4_, CoSO_4_ were prepared before the assay. To minimize oxidation of Cr^2+^ ions, aqueous solution (5 mmol/L) of CrCl_2_ was prepared with deaerated water within 1 min before addition to a luminescent reaction mixture.

Working solutions of H_2_O_2_ (2.8, 14, 28 and 56 mmol/L) were prepared by dilution of 30% H_2_O_2_ solution and the concentration was confirmed by the measurement of absorbance at 240 nm using a molar extinction coefficient of 43.6/mol cm [44]. Stock solution of EGTA (100 mmol/L) was prepared in PBS with pH adjusted to 8.0 with 5 mol/L NaOH and stored at room temperature in the dark for no longer than 3 months. EGTA working solutions (concentrations of 1, 5, 10 and 20 mmol/L) were obtained by appropriate dilution of EGTA stock solution with water. Catalase and SOD were dissolved in PBS to an activity of 10 U/µL. Phenolic acids (ferulic, chlorogenic and caffeic acids) were dissolved in PBS to concentrations of 0.1, 0.2 and 1 mmol/L. Solutions of DMSO (concentrations of 20, 40, 80 and 120 mmol/L), NaN_3_ (20 mmol/L), mannitol (20 mmol/L), EDTA (10 mmol/L), citric acid (10 mmol/L) and sodium ascorbate (25 mmol/L) were prepared in PBS freshly before the assay. 

### 4.2. Light Emission from Fe^2+^-EGTA-H_2_O_2_ System

The chemiluminescence was measured with a multitube luminometer (AutoLumat Plus LB 953, Berthold, Germany) equipped with a Peltier-cooled photon counter (spectral range from 380 to 630 nm) to ensure high sensitivity and low and stable background noise signal. Twenty µL of 10 mmol/L EGTA solution was added to the tube (Lumi Vial Tube, 5 mL, 12 × 75 mm, Berthold Technologies, Bad Wildbad, Germany) containing 940 µL of PBS. Then 20 µL of 5 mmol/L solution of FeSO_4_ was added and after gentle mixing the tube was placed in the luminometer chain and incubated for 10 min in the dark at 37 °C. Then 100 µL of 28 mmol H_2_O_2_ solution was added by an automatic dispenser and the total light emission (expressed in RLU) was measured for 120 s. The final concentrations of FeSO_4_, EGTA and H_2_O_2_ in the reaction mixture were 92.6, 185.2 and 2.6 mmol/L respectively. Control systems included: incomplete system I (Fe^2+^-H_2_O_2_ in PBS); incomplete system II (EGTA-H_2_O_2_ in PBS); H_2_O_2_ in PBS; Fe^2+^ and EGTA without H_2_O_2_ (Fe^2+^-EGTA-H_2_O in PBS); and medium alone (H_2_O in PBS) (Table 3). These experiments were also performed with 2- and 10-times lower and 2-times higher concentrations of FeSO_4_, EGTA and H_2_O_2_ (the ratio of molar concentrations of compounds was always the same). 

### 4.3. Effect of Iron and EGTA Replacement by other Divalent Cations and Metal Chelators on Light Emission from Fe^2+^-EGTA-H_2_O_2_ System

In these experiments we checked whether replacement of Fe^2+^ and EGTA with other divalent cations (Cr^2+^, Co^2+^, Mn^2+^, Cu^2+^) and metal chelators (EDTA and sodium citrate) can change the light emission from the Fe^2+^-EGTA-H_2_O_2_ system. The Fenton system was 92.6 µmol/L Fe^2+^—185.2 µmol/L EGTA—2.6 mmol/L H_2_O_2_ and Fe^2+^ was replaced by the same concentrations of the aforementioned cations. In another series of experiments EGTA was replaced by the same concentrations of EDTA or sodium citrate and the total light emission was measured as described. The design of these experiments together with appropriate controls is shown in Table 3.

### 4.4. Determining the Effect of Reactive Oxygen Species Scavengers and Selected Phenolic Acids on Light Emission from Fe^2+^-EGTA-H_2_O_2_ System

To determine what ROS are involved in the UPE of 92.6 µmol/L Fe^2+^—185.2 µmol/L EGTA—2.6 mmol/L H_2_O_2_ system, 20 µL solution of ROS scavenger was added to the luminometer tube containing FeSO_4_ and EGTA in PBS and incubated for 10 min at 37 °C in the dark and then 100 µL of H_2_O_2_ solution was injected and the total light emission was measured for 2 min. Controls included: full system without ROS scavenger (Fe^2+^-EGTA-H_2_O_2_ in PBS); incomplete system I (Fe^2+^-H_2_O_2_ in PBS); incomplete system I with ROS scavenger (Fe^2+^-ROS scavenger-H_2_O_2_ in PBS); Fe^2+^ and EGTA without H_2_O_2_ (Fe^2+^-EGTA-H_2_O in PBS); Fe^2+^ and EGTA and ROS scavenger without H_2_O_2_ (Fe^2+^-EGTA-ROS scavenger-H_2_O in PBS) (Table 4). 

The following ROS scavengers were used SOD—an O_2_·^−^ scavenger (final activity of 0.185 U/µL), catalase—an H_2_O_2_ scavenger (final activity of 0.185 U/µL), DMSO—a potent ^•^OH scavenger [45] (final concentrations of 0.37 mmol/L to 2.22 mmol/L), mannitol—an ^•^OH scavenger [46] (final concentration of 0.37 mmol/L), NaN_3_—a O_2_(^1^Δ_g_) scavenger [47] (final concentration of 0.37 mmol/L) and sodium ascorbate (final concentration of 0.46 mmol/L). In another series of experiments the effects of three phenolic acids (ferulic, chlorogenic and caffeic acids, final concentrations in the reaction mixture from 0.09 mmol/L to 0.87 mmol/L) on total light emission from Fe^2+^-EGTA-H_2_O_2_ system were studied. The design of these tests and control samples were the same as in the case of ROS scavengers (Table 4). In each series of experiments (repeated at least four times) one ROS scavenger or one phenolic acid was tested. The inhibitory effect of ROS scavengers or phenolic acids on light emission was expressed as percent inhibition (%I) calculated according to the formula: %I = [(A − B)/(A − C)] × 100% where A, B and C are the total light emission from Fe^2+^-EGTA-H_2_O_2_, Fe^2+^-EGTA-studied compound-H_2_O_2_, and medium (H_2_O in PBS), respectively.

### 4.5. Statistical Analysis 

Results (total light emission or % inhibition of light emission) were expressed as mean (standard deviation) and median and interquartile range (IQR). The comparisons between total light emission from Fe^2+^-EGTA-H_2_O_2_ system and light emission from corresponding samples of modified system (e.g., incomplete system, system with addition of ROS scavengers or phenolic acids, system based on other divalent cations or chelating agents, medium alone) were analyzed with independent-samples (unpaired) *t*-test or Mann–Whitney *U* test depending on data distribution which was tested with Kolmogorov-Smirnov-Liliefors test. The Brown-Forsythe test for analysis of the equality of the group variances was used prior to the application of the unpaired *t*-test and if variances were unequal, the Welch’s *t*-test was used instead of the standard *t*-test. The comparisons of % inhibition of light emission caused by ROS scavengers and phenolic acids were performed in the same way. A *p* value < 0.05 was considered significant.

## 5. Conclusions 

We found that Fenton system composed of Fe^2+^-EGTA-H_2_O_2_ emits light within the range from 380 nm to 630 nm. The UPE of Fe^2+^-EGTA-H_2_O_2_ depends upon ^•^OH and O_2_^−^ and could be attributed to ^•^OH—induced cleavage of ether bond in the backbone structure of EGTA with consequent formation of ^3^(R=C)* and photons emission. Plant phenolic acids with known antioxidant properties (caffeic, chlorogenic and ferulic acids) and ascorbic acid significantly quenched UPE what suggests possible application of this phenomenon as the assay for evaluation of antioxidant activities of various phytochemicals. However, further studies involving optimization of the Fenton system parameters, time of UPE measurement, control tests with other types of antioxidants and validation are necessary before the successful development of this assay. 

## Figures and Tables

**Table 1:** Light emission from Fe^2+^-EGTA-H_2_O_2_ system. Effect of increasing concentrations of EGTA-modified Fenton system under stable ratio of Fe^2+^ to EGTA to H_2_O_2_ molar concentration conditions.

Total Light Emission [RLU]						
Experiment	Sample Composition in Phosphate Buffered Saline (pH = 7.4)					
	Fe^2+^-EGTA-H_2_O_2_	Fe^2+^-H_2_O_2_	EGTA-H_2_O_2_	H_2_O_2_	Fe^2+^-EGTA-H_2_O	H_2_O
A (*n* = 4)	1351 ± 178 * (1343;212)	594 ± 58 (602;86)	612 ± 68 (607;70)	543 ± 37 (547;26)	578 ± 70 (574;66)	530 ± 38 (523;32)
B (*n* = 5)	1533 ± 76 * (1552;139)	522 ± 36 (515;53)	507 ± 14 (512;13)	522 ± 43 (525;34)	489 ± 9 (486;14)	472 ± 12 (472;15)
C (*n* = 10)	4319 ± 755 *† (4355;1127)	645 ± 100 ** (628;117)	600 ± 80 (594;80)	565 ± 63 (556;74)	545 ± 77 (515;95)	521 ± 64 (497;70)
D (*n* = 8)	6278 ± 502 *† (6070;296) ‡	609 ± 77 ** (610;62)	549 ± 55 (559;85)	526 ± 50 (507;71)	495 ± 40 (495;53)	497 ± 38 (488;66)

*n*—number of separate experiments. Total light emission was measured for 2 min just after automatic injection of 100 µL of H_2_O_2_ solution or distilled water. Final sample volume 1080 µL. Results expressed as mean and standard deviation and (median; interquartile range). The concentrations of Fe^2+^, EGTA and H_2_O_2_ were 9.3, 18.5 and 0.26 mmol/L for experiment A; 46.3, 92.6 and 1.3 mmol/L for B; 92.6, 185.2 and 2.6 mmol/L for C; and 185.2, 370.4 and 5.2 mmol/L for experiment D, respectively. The ratio of Fe^2+^ to EGTA to H_2_O_2_ molar concentrations in the Fenton reaction system was the same for all experiments (1:2:28.1). * vs. all corresponding values of the same experiment, *p* < 0.001; ** vs. corresponding values of EGTA-H_2_O_2_, H_2_O_2_ alone, Fe^2+^-EGTA-H_2_O and H_2_O alone, *p* < 0.05; † vs. corresponding value of experiment A and B, *p* < 0.01; ‡ vs. corresponding value of experiment C, *p* < 0.01.

**Table 2:** Inhibition of light emission from Fe^2+^-EGTA-H_2_O_2_ by selected phenolic acids.

Compound Concentration (µmol/L)	% Inhibition of Light Emission from Fe^2+^-EGTA-H_2_O_2_ System		
	Ferulic Acid	Chlorogenic Acid	Caffeic Acid
87	90 ± 4 (90;7) *	90 ± 5 (90;8)	97 ± 2 (97;3)
174	90 ± 3 (90;5) *	94 ± 3 (94;5)	98 ± 1 (98;1)
870	90 ± 5 (88;9) *	91 ± 5 (88;8) *	98 ± 3 (98;2)

Phenolic acid was mixed with EGTA and Fe^2+^ and then H_2_O_2_ was automatically injected with subsequent measurement of total light emission for 2 min. Results obtained from at least four separate experiments. * vs. corresponding concentration of caffeic acid, *p* < 0.05.

**Table 3:** Design of experiments on light emission from Fe^2+^-EGTA-H_2_O_2_ system.

No	Sample	Volumes of Working Solutions Added to Luminometer Tube (µL)				
		A	B *	C **	D	E
		PBS	EGTA	FeSO_4_	H_2_O_2_	H_2_O
1	Complete system	940	20	20	100	-
2	Incomplete system I	960	-	20	100	-
3	Incomplete system II	960	20	-	100	-
4	H_2_O_2_ alone	980	-	-	100	-
**Additional controls**						
5	Fe^2+^-EGTA without H_2_O_2_	940	20	20	-	100
6	Medium alone	980	-	-	-	100

Working solutions were mixed in alphabetical order. A—sterile phosphate buffered saline (PBS) (pH = 7.4) without divalent cations; B—10 mmol/L aqueous solution of EGTA; C—5 mmol/L aqueous solution of FeSO_4_. Then after gentle mixing the tube was placed into luminometer chain, incubated for 10 min at 37 °C and then 28 mmol/L H_2_O_2_ (D) or water (E) was automatically injected with dispenser and total light emission was measured for 2 min. * in certain experiments the same concentration of EDTA or citric acid solution was added instead of EGTA solution. ** in certain experiments the same concentration of CuSO_4_, CoSO_4_, MnSO_4_ or CrCl_2_ solution was added instead of FeSO_4_ solution. Some experiments were performed with 2- and 5-times lower and 2-times higher concentrations of FeSO_4_, EGTA and H_2_O_2_.

**Table 4:** Design of experiments on the effect of reactive oxygen scavengers and selected phenolic acids on light emission from Fe^2+^-EGTA-H_2_O_2_ system.

No	Sample	Volumes of Working Solutions Added to Luminometer Tube (µL)						
		A	B	C	D	E	F	G
		PBS	Polyphenol	EGTA	FeSO_4_	ROS Scavenger	H_2_O_2_	H_2_O
1	Complete system	940	-	20	20	-	100	-
2	Complete system + polyphenol	-	940	20	20	-	100	-
3	Complete system + ROS scavenger	920	-	20	20	20	100	-
4	Incomplete system I	960	-	-	20	-	100	-
5	Incomplete system I + polyphenol	20	940	-	20	-	100	-
6	Incomplete system I + ROS scavenger	940	-	-	20	20	100	-
**Additional controls**								
7	Fe^2+^-EGTA without H_2_O_2_	940	-	20	20	-	-	100
8	Fe^2+^-EGTA without H_2_O_2_ + polyphenol	-	940	20	20	-	-	100
9	Fe^2+^-EGTA without H_2_O_2_ + ROS scavenger	920	-	20	20	20	-	100

Working solutions were mixed in alphabetical order. A—sterile phosphate buffered saline (PBS) (pH = 7.4) without divalent cations; B—Polyphenol solution in PBS (ferulic acid, chlorogenic acid or caffeic acid, concentrations from 0.1 mmol/L to 1 mmol/L); C—10 mmol/L aqueous solution of EGTA; D—5 mmol/L aqueous solution of FeSO_4_; E—Solution of ROS scavenger in PBS (10 U/µL SOD, 10 U/µL catalase, 20 mmol/L mannitol, 20 mmol/L NaN_3_, 25 mmol/L sodium ascorbate or 20 mmol/L to 120 mmol/L DMSO). Then after gentle mixing the tube was placed into luminometer chain, incubated for 10 min at 37 °C and then 28 mmol/L H_2_O_2_ (F) or water (G) was automatically injected with dispenser and total light emission was measured for 2 min.

## Abbreviations

UPE	ultra-weak photon emission
^•^OH	hydroxyl radical
ROS	reactive oxygen species
EDTA	ethylenediaminetetraacetic acid, disodium salt
EGTA	ethylene glycol-bis(β-aminoethyl ether)-*N*,*N*,*N*′,*N*′-tetraacetic acid
RLU	relative light units
SOD	superoxide dismutase
DMSO	dimethyl sulfoxide
O_2_^−^	superoxide radical
O_2_(^1^Δ_g_)	singlet oxygen
^3^(R=C)*	triplet excited carbonyl groups

## Appendix A. Chemical Compounds Studied in This Article

Caffeic acid (PubChem CID: 689043); Chlorogenic acid (PubChem CID: 1794427); EDTA disodium salt (PubChem CID: 8758); EGTA (PubChem CID: 6207); Ferulic acid (PubChem CID: 445858); Sodium citrate (PubChem CID: 6224).
